# Comparison of Dural Venous Sinus Volumes Before and After Flight in Astronauts With and Without Spaceflight-Associated Neuro-Ocular Syndrome

**DOI:** 10.1001/jamanetworkopen.2021.31465

**Published:** 2021-10-27

**Authors:** Mark J. Rosenberg, Michael A. Coker, James A. Taylor, Milad Yazdani, M. Gisele Matheus, Christopher K. Blouin, Sami Al Kasab, Heather R. Collins, Donna R. Roberts

**Affiliations:** 1 Department of Neurology, Medical University of South Carolina, Charleston; 2 Department of Radiology and Radiological Science, Medical University of South Carolina, Charleston; 3 Department of Neurosurgery, Medical University of South Carolina, Charleston

## Abstract

**Importance:**

Spaceflight-associated neuro-ocular syndrome (SANS) occurs in 40% to 60% of National Aeronautics and Space Administration (NASA) International Space Station (ISS) astronauts who present postflight with ophthalmological findings and elevated intracranial pressure. The etiology of SANS is unknown; it is hypothesized that venous outflow congestion from the head and neck occurs because of microgravity, which is supported by the finding of internal jugular vein stagnant flow and thrombosis in some astronauts, but the impact on intracranial dural venous sinus structures remains unknown.

**Objectives:**

To clarify the potential risk of retrograde extension of clot intracranially among astronauts with internal jugular venous thrombosis by evaluating intracranial venous structures following spaceflight and to assess for any association between intracranial venous congestion and SANS.

**Design, Setting, and Participants:**

This retrospective cohort study of all NASA astronauts who had undergone magnetic resonance (MR) venography at the time of the study included quantitative and qualitative assessments of the intracranial venous system on preflight and postflight MR venograms. Data were collected a mean (SD) of 525.8 (187.5) days before spaceflight and 2.0 (1.5) days after return to Earth. A semiautomated segmentation of the venogram images was used, which was then compared with a neuroradiologist’s assessment.

**Exposures:**

A mean (SD) 184.3 (66.0) days of ISS spaceflight missions.

**Main Outcomes and Measures:**

Dural venous sinus volumes before and after spaceflight.

**Results:**

A total of 12 astronauts (2 [16.67%] women; 10 [83.33%] men), with a mean (SD) age of 47.8 (5.8) years, were included. Overall, 4 astronauts (33.33%) met the diagnostic criteria for SANS. No dural venous sinus thrombosis was detected for any astronaut. Astronauts with SANS had significantly greater median (range) preflight to postflight increases in volume vs astronauts without SANS for all 3 venous sinus structures: superior sagittal sinus (13.40% [8.70% to 17.47%] vs −2.66% [−15.84% to 5.31%,]; *P* = .004), right transverse/sigmoid sinus (17.15% [7.63% to 30.08%] vs 0.77% [−14.98% to 15.12%]; *P* = .02), and left transverse/sigmoid sinus (9.40% [5.20% to 15.50%] vs −1.40% [−14.20% to 12.50%]; *P* = .03). There was a positive correlation between the neuroradiologist’s evaluation and the semiautomated method for the superior sagittal sinus (*r*_pb_ = 0.64; *P* = .02) and the right transverse/sigmoid sinus (*r*_pb_ = 0.58; *P* = .050).

**Conclusions and Relevance:**

These findings, in conjunction with the growing body of evidence of abnormal blood flow dynamics during spaceflight, suggest an association between intracranial venous congestion and SANS. Thus, there is an implication that individuals with increased venous sinus compliance may be at increased risk of developing SANS. These findings should be confirmed in a larger astronaut population and may contribute to understanding disorders of intracranial venous outflow on Earth.

## Key Points

**Question:**

Given that thrombosis and abnormal internal jugular venous flow have been documented in astronauts, does a retrograde extension of clot occur intracranially, and is abnormal dural venous sinus flow associated with spaceflight associated neuro-ocular syndrome (SANS)?

**Findings:**

In this cohort study of 12 astronauts, there was no evidence of intracranial dural venous sinus thrombosis. Astronauts with SANS had significantly greater preflight to postflight increases in intracranial dural venous volumes than astronauts without SANS.

**Meaning:**

The finding of an association between intracranial venous congestion and SANS suggests that aberrant venous flow may play a role in the development of SANS and astronauts with increased venous sinus compliance may be at increased risk.

## Teaser

This cohort study clarifies the risk of internal jugular venous thrombosis among astronauts by evaluating the intracranial venous structures following spaceflight and assesses associations between intracranial venous congestion and spaceflight-associated neuro-ocular syndrome (SANS).

## Introduction

Spaceflight-associated neuro-ocular syndrome (SANS) occurs in approximately 40% to 60% of National Aeronautics and Space Administration (NASA) International Space Station (ISS) astronauts who present post flight with altered visual acuity, areas of injury to the retina, globe flattening, optic disk edema, and mildly elevated intracranial pressures (ICPs).^1^ The etiology of SANS is unknown, but a leading hypothesis is congestion of venous outflow from the head and neck because of microgravity-induced cephalad fluid shifts. This was supported by detection of stagnant flow by Doppler ultrasound^2^ and, in 2 cases, thrombosis in the internal jugular veins of ISS astronauts.^3^ Considering the unknown risk of thrombus embolism and retrograde extension to the intracranial veins, a decision was made to provide anticoagulation treatment to the 1 astronaut who was diagnosed with thrombosis while aboard the ISS.^3^ Treatment was carried out until 4 days prior to return to Earth.^3^

The association of internal jugular vein stagnant flow and thrombosis with intracranial venous pressure as well as intracranial and intraocular pressure remains unknown. In this study, we conducted retrospective quantitative and qualitative assessments of the intracranial venous system in a cohort of NASA astronauts who had undergone magnetic resonance images (MRI) before and after spaceflight missions to the ISS.

## Methods

### Participants

At the time of data acquisition for this study, all NASA astronauts who had undergone MR venograms before and after ISS missions were included as participants in this study. While MRIs have been performed before and after spaceflight on all astronauts routinely, per NASA medical requirements since 2009, only recently has intracranial MR venography been included as part of the NASA medical operations protocol. Based on published guidelines,^1^ astronauts who met the diagnostic criteria for SANS postflight were included in the SANS group, while the remainder were included in the non-SANS group.

The MRI scans along with demographic data for this study were obtained from the NASA Lifetime Surveillance of Astronaut Health Program. The study was approved by the institutional review boards at the NASA Johnson Space Center and the Medical University of South Carolina. All astronauts signed written informed consent for use of their data, and the manuscript was reviewed by the NASA Lifetime Surveillance of Astronaut Health Office to ensure astronaut anonymity. The Strengthening the Reporting of Observational Studies in Epidemiology (STROBE) reporting guideline was followed.

### Imaging Protocols and Image Processing

MRI was performed on a 3T Siemens Verio scanner and included an MR venogram and a volumetric T1-weighted magnetization-prepared rapid acquisition of gradient-echo sequence. MR venograms were performed using a flow sensitive technique without administration of intravenous contrast with the following parameters: 0.5 × 0.5 × 1.6 mm^3^ voxels; repetition time, 21 milliseconds; echo time, 5.42 milliseconds; flip angle, 30°. The following parameters were used for the structural T1-weighted sequences: 0.9 × 0.5 × 0.45 mm^3 ^voxels; repetition time, 1900 milliseconds; echo time, 2.32 milliseconds; flip angle, 9°.

**Figure 1.**
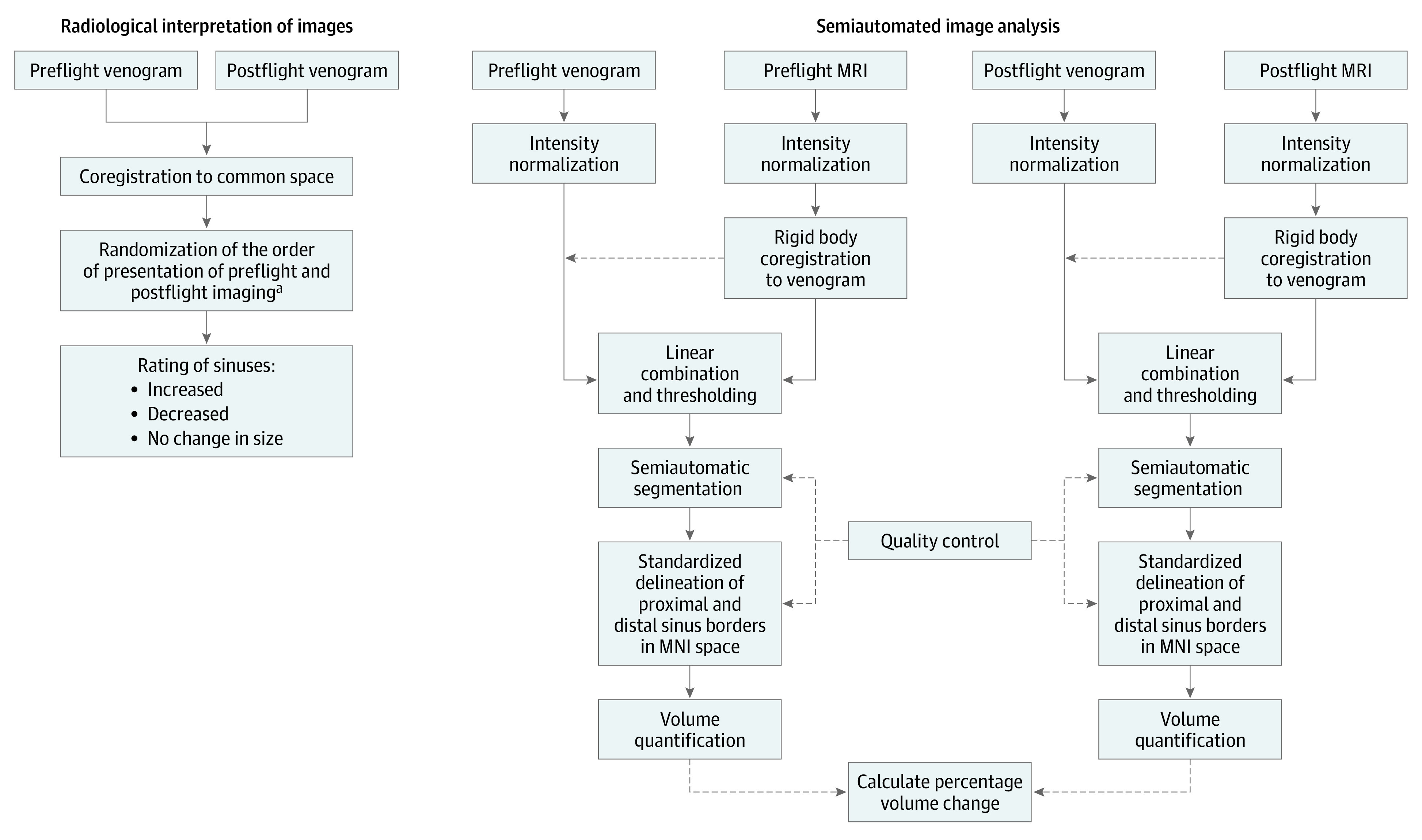
Image Analysis Workflow MNI indicates Montreal Neurological Institute; MRI, magnetic resonance imaging. ^a^Radiologist was masked to astronauts’ spaceflight-associated neuro-ocular syndrome status.

Venous structures (ie, superior sagittal sinus, left and right transverse/sigmoid sinuses) were segmented using a semiautomated pipeline, and preflight to postflight percentage changes in venous volumes were calculated (Figure 1). The venograms were processed to enhance contrast before segmentation. The images were corrected for intensity in homogeneity artifact using the N4BiasFieldCorrection program from the Advanced Normalization Tools toolbox (version 2.3.4).^4^ Then, each astronauts’ structural images were aligned to their corresponding MR venograms at each corresponding preflight and postflight time using spm_coreg and spm_reslice^5^ from the Statistical Parametric Mapping (SPM) version 12 software suite. Once aligned, the structural images were subtracted from the venograms to provide a greater contrast between the tissue background and the bright sinuses, a feature not present in the structural images. Voxels in the difference images were then thresholded at 14% of maximum brightness, leaving only the sinus features.

Binary masks of the sinus regions were then obtained from the processed venogram images using itk-SNAP^6^ version 3.2 for semiautomatic segmentation.^7^ To ensure that the extent of the sinus masks was the same, sinus image volume anterior to Y = 45 mm or inferior to Z = −65 mm in Montreal Neurological Institute (MNI) 152 standard space was excluded. To do this, binary masks of these regions were made in MNI space. Then, they were moved to each venogram image set and used as a mask. This alignment was composed of a nonlinear transformation made by fnirt (FMRIB Software Library^8^ version 6.0.3) from MNI space to each participant’s structural image and a rigid body registration made by spm_coreg in SPM.^9,10,11^

The sinus masks were divided in a similar manner into right transverse/sigmoid sinus, left transverse/sigmoid sinus, and superior sagittal sinus. Sinus volume superior of Z = −15.5 mm was considered superior sagittal; sinus volume inferior of Z = −15.5 mm and right of X = 0.5 mm was considered right transverse; sinus volume inferior of Z = −15.5 mm and left of X = 0.5 mm was considered left transverse.

Quality control was then performed by a neuroradiologist (D.R.R.) with 15 years of experience to ensure the masks accurately delineated each sinus. The sinus volumes were then found from the masks using MATLAB version R2019b (Mathworks).

### Radiologist Interpretation of Images

To evaluate whether the preflight to postflight changes in size of the venous structures were clinically visible, the paired preflight and postflight venogram images were presented in a randomized order to a board-certified neuroradiologist (M.Y.) with 6 years of experience (Figure 1). For each image pair, the radiologist rated the superior sagittal sinus and left and right transverse/sigmoid sinuses as either increased, decreased, or no change in size between the 2 image sets. The radiologist was masked to each astronaut’s SANS status.

### Statistical Analysis

Differences in the preflight to postflight percentage change in venous sinus volumes were evaluated with Mann-Whitney *U* tests and summarized with medians and ranges. Point-biserial correlations (*r*_pb_) were run to examine the association between the reader’s evaluation of venous sinus volume changes and the semiautomated method. Spearman rank order correlation (*r*_s_) was used to assess the correlation between the number of days between return and postflight MRI and the dural venous volume changes. Statistical significance was considered at the α = .05 threshold, with 2-sided *P* values reported. Data analysis was conducted in SPSS statistical software version 25 (IBM Corp).

## Results

At the time of data acquisition for this study, 12 NASA astronauts (2 [16.67%] women; 10 [83.33%] men; mean [SD] age, 47.8 [5.8] years) had undergone intracranial MR venography before and after ISS missions (mean [SD] duration, 184.3 [66.0] days) and therefore were included as participants in this study (Table). Imaging was performed a mean (SD) of 525.8 (187.5) days prior to spaceflight and mean (SD) 2.0 (1.5) days after return to Earth.

**Table.** Demographic Characteristics

Characteristic	Astronauts	
	With SANS (n = 4)	Without SANS (n = 8)
Age, mean (SD), y	51.3 (2.8)	46.0 (6.2)
Women, No. (%)	1 (25.0)	1 (12.5)
Men, No. (%)	3 (75.0)	7 (87.5)
Mission duration, mean (SD), d	241.5 (87.5)	155.6 (27.5)

Abbreviation: SANS, spaceflight-associated neuro-ocular syndrome.

Based on published guidelines,^1^ 4 of the 12 astronauts (33.33%) met the diagnostic criteria for SANS postflight, including globe flattening in 3 (75.00%), choroidal folds in 4 (100%), and optic disc edema in 3 (75.00%), and were included in the SANS group. The other 8 astronauts were included in the non-SANS group.

**Figure 2.**
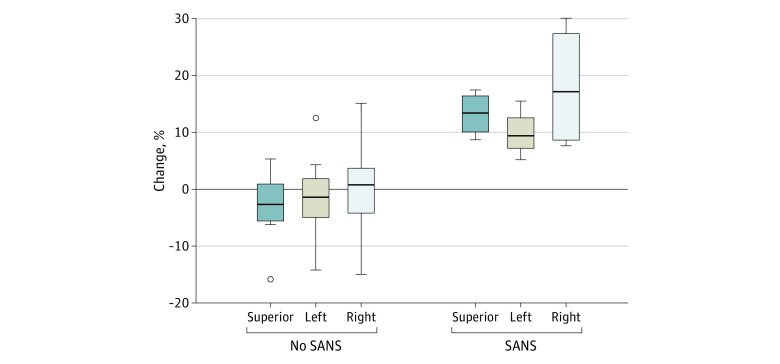
Preflight to Postflight Percentage Changes in Venous Sinus Volumes for the Superior Sagittal Sinus and Left and Right Transverse/Sigmoid Sinuses for Astronauts With and Without Spaceflight-Associated Neuro-Ocular Syndrome (SANS) The boxes indicate the IQR, with the line in each box representing the median and the edges representing the limits of the IQR. The whiskers indicate 1.5 times the IQR, and the open circles indicate data points from 1.5 to 3.0 times the IQR.

**Figure 3.**
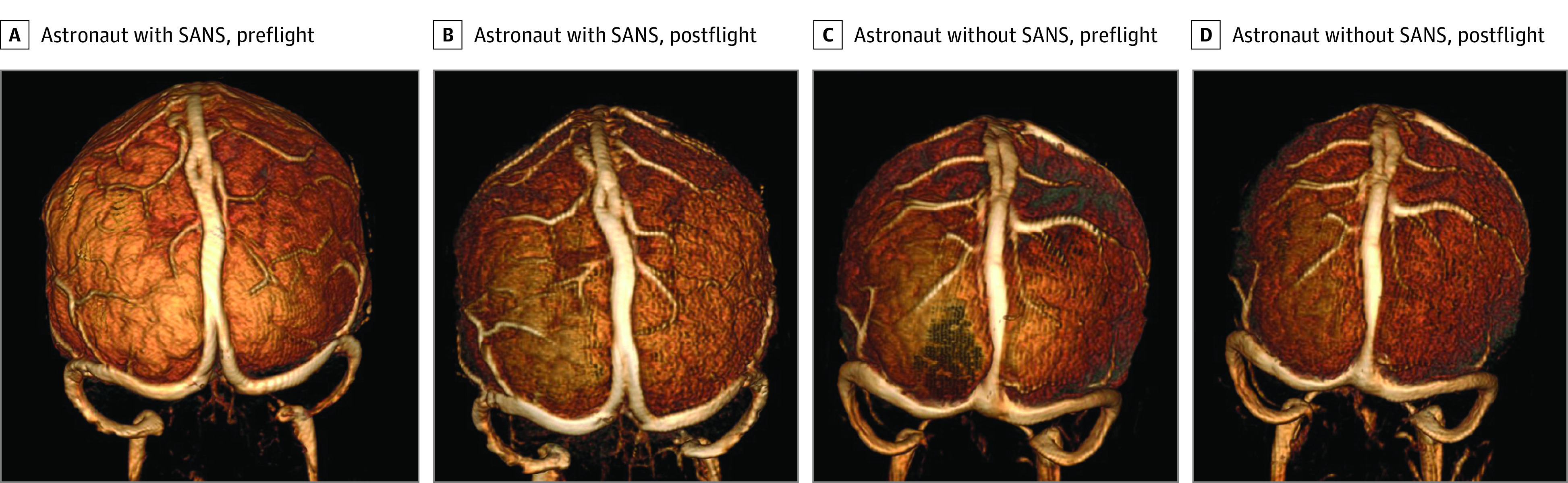
Three-Dimensional Reconstructions of the Preflight and Postflight Venograms for an Astronaut with Spaceflight-Associated Neuro-Ocular Syndrome (SANS) and an Astronaut Without SANS These images were created using RadiAnt DICOM Viewer, version 2020.2.2 (Medixant).

On MR venography, there was no evidence of dural venous sinus or cortical vein thrombosis for any of the astronauts before or after flight. Astronauts with SANS had significantly greater median (range) preflight to postflight increases in volume than astronauts without SANS for all 3 venous sinus structures (Figure 2 and Figure 3): superior sagittal sinus (13.40% [8.70% to 17.47%] vs −2.66% [−15.84% to 5.31%]; *P* = .004), right transverse/sigmoid sinus (17.15% [7.63% to 30.08%] vs 0.77% [−14.98% to 15.12%]; *P* = .02), and left transverse/sigmoid sinus (9.40% [5.20% to 15.50%] vs −1.40% [−14.20% to 12.50%]; *P* = .03). There were no significant correlations between the number of days between return and postflight MRI and the volume change in the right transverse/sigmoid sinus (*r*_s_ = −0.02, *P* = .96), left transverse/sigmoid sinus (*r*_s_ = 0.20, *P* = .53), or superior sagittal sinus (*r*_s_ = 0.09, *P* = .78).

There was only 1 instance of a no change reading by the neuroradiologist, so that value was excluded from statistical analysis. There was a positive correlation between the reader’s evaluation and the automatic method for the superior sagittal sinus (*rpb* = 0.64, *P* = .02) and the right transverse/sigmoid sinus (*rpb* = 0.58, *P* = .050). There was a similar finding for the left transverse/sigmoid sinus; however, it did not reach statistical significance (*rpb* = 0.59, *P* = .06).

## Discussion

In this study, NASA astronauts who presented clinically with SANS had enlargement of the dural venous sinuses consistent with intracranial venous congestion. In addition, our study found a correlation between semiautomated and experienced neuroradiologist assessments in evaluating venous sinus congestion in this population.

SANS is considered by NASA to be one of the highest priority risks for human spaceflight.^12,13^ A leading hypothesis for the development of SANS is that in microgravity, loss of gravitational hydrostatic pressure leads to an upward shift of fluids from the lower body, with resultant venous congestion in the head and neck.^13,14^ However, it is unclear whether the venous congestion acts locally at the level of the intraorbital optic nerve and globe leading directly to the optic disc edema and areas of injury to the retina seen in SANS^15^ or, alternatively, head and neck venous congestion obstructs venous outflow from the cranium, leading to intracranial hypertension, which is then transmitted along the optic nerve sheath, ultimately resulting in optic disc edema.^1,16^ Based on this second hypothesized mechanism for SANS, idiopathic intracranial hypertension (IIH) has been considered a terrestrial model for SANS.^16^ IIH is a disorder of cerebrospinal fluid homeostasis in which patients present clinically with signs and symptoms of increased ICP, including vision changes, headaches, and papilledema.^17^ While the exact pathophysiology of IIH is unknown, venous hypertension and outflow obstruction have been proposed as primary mechanisms.^18^ Our findings of venous congestion in intracranial venous sinuses supports the theory that SANS represents a global ICP pathology rather than an isolated ocular pathology. We hypothesize that venous congestion leads to increased ICP, which manifests in optic disk edema.

On Earth, venous outflow obstruction can result from blockage of the dominant transverse sinus by intraluminal structures, such as prominent arachnoid granulations, chronic thrombus, fenestrations, and congenital septations, which appear at MR venography as focal areas of dural venous sinus narrowing.^19^ At invasive manometry, pressure gradients of greater than 10 mm Hg can be present across these focal stenoses,^20^ and treatment with stent placement across the stenoses reduces the pressure gradient, normalizing ICP.^21,22^ Another appearance in patients with IIH at MR venography is long-segment transverse sinus narrowing, thought to result from extrinsic compression of the transverse sinus in the setting of elevated ICP.^23^ Therefore, on Earth, the level of venous outflow obstruction occurs within the cranium because of intrinsic and/or extrinsic factors. For NASA astronauts with SANS, instead of narrowing, we found enlargement of the dural venous sinuses, which suggests the level of obstruction is further downstream, outside the cranium. We hypothesize dural venous sinus congestion in SANS results from the lack of normal drainage, facilitated on Earth by gravity, through the internal jugular veins and vertebral venous plexuses, as was recently documented in some ISS astronauts.^2^

Unlike most veins, the dural venous sinuses are rigid structures that resist deformation despite large physiological variations in surrounding ICP.^24,25^ They are triangular in shape, formed from the tough periosteal and meningeal layers of dura, and are protected anatomically by attachment to the inner table of the skull.^25^ Abnormal dural venous sinus compliance is considered by some to be a key factor in the development of IIH, as laxity of the dural venous sinus walls would allow narrowing at times of high ICP, thereby contributing further to venous outflow obstruction.^24^ Here, distention of the walls of the dural venous sinuses in astronauts with SANS may reflect venous sinus laxity. Laxity may be a risk factor for the development of SANS, as dural venous sinus rigidity in astronauts without SANS may serve to resist the development of intracranial venous congestion.

An alternative explanation for our findings may be that enlargement of the dural sinuses in astronauts with SANS occurs after return to Earth, prior to the postflight MRI. Imaging was performed in all of the astronauts within 5 days after landing, and we found no association with time to postflight imaging. However, compression of the venous system has been documented in patients with IIH, which resolves after cerebrospinal fluid diversion,^23^ and the possibility that a reduction in ICP from in-flight levels on return to Earth could have resulted in expansion of the intracranial venous system needs further study. As dural sinus pressure is always maintained below cerebrospinal fluid pressure, because of the waterfall effect occurring at the level of the bridging veins, enlargement of the dural sinuses may reflect an abrupt ICP normalization on return to a 1 gravity environment, with a rebound cerebrospinal fluid overdrainage.^26,27^ Supporting this explanation, abrupt changes in ICP have been documented when alternating between microgravity and hypergravity during parabolic flight.^28^ Arguing against this possibility is the moderately elevated opening pressures of as much as 28.5 cm of water documented post flight, months after return to Earth, in astronauts with SANS.^1^

### Limitations

This study has limitations, including the small number of astronauts and the lack of invasive ICP and intravenous pressure correlation. These data would be helpful in determining the physiological significance of the change in dural venous sinus volumes that we document here. An additional limitation is the lack of data concerning any potential SANS countermeasure usage among the astronaut cohort.

## Conclusions

Our study, in conjunction with the growing body of evidence of abnormal blood flow dynamics with venous outflow stagnation during spaceflight, suggests an association between intracranial venous congestion and SANS. The implication thus exists that individuals with increased venous sinus compliance may be at increased risk of developing SANS. These findings should be confirmed in a larger astronaut population. These results underscore the need for further studies to determine whether changes in the venous sinus volumes documented here are clinically relevant and whether venous congestion contributes to the development of SANS or headaches among astronauts. Additionally, these results may provide insight into terrestrial disorders of intracranial venous congestion and cerebrospinal fluid homeostasis.
